# Identifying Data Quality Dimensions for Person-Generated Wearable Device Data: Multi-Method Study

**DOI:** 10.2196/31618

**Published:** 2021-12-23

**Authors:** Sylvia Cho, Chunhua Weng, Michael G Kahn, Karthik Natarajan

**Affiliations:** 1 Department of Biomedical Informatics Columbia University New York, NY United States; 2 Department of Pediatrics University of Colorado Anschutz Medical Campus Aurora, CO United States

**Keywords:** patient-generated health data, data accuracy, data quality, wearable device, fitness trackers, qualitative research

## Abstract

**Background:**

There is a growing interest in using person-generated wearable device data for biomedical research, but there are also concerns regarding the quality of data such as missing or incorrect data. This emphasizes the importance of assessing data quality before conducting research. In order to perform data quality assessments, it is essential to define what data quality means for person-generated wearable device data by identifying the data quality dimensions.

**Objective:**

This study aims to identify data quality dimensions for person-generated wearable device data for research purposes.

**Methods:**

This study was conducted in 3 phases: literature review, survey, and focus group discussion. The literature review was conducted following the PRISMA (Preferred Reporting Items for Systematic Reviews and Meta-Analyses) guideline to identify factors affecting data quality and its associated data quality challenges. In addition, we conducted a survey to confirm and complement results from the literature review and to understand researchers’ perceptions on data quality dimensions that were previously identified as dimensions for the secondary use of electronic health record (EHR) data. We sent the survey to researchers with experience in analyzing wearable device data. Focus group discussion sessions were conducted with domain experts to derive data quality dimensions for person-generated wearable device data. On the basis of the results from the literature review and survey, a facilitator proposed potential data quality dimensions relevant to person-generated wearable device data, and the domain experts accepted or rejected the suggested dimensions.

**Results:**

In total, 19 studies were included in the literature review, and 3 major themes emerged: device- and technical-related, user-related, and data governance–related factors. The associated data quality problems were incomplete data, incorrect data, and heterogeneous data. A total of 20 respondents answered the survey. The major data quality challenges faced by researchers were completeness, accuracy, and plausibility. The importance ratings on data quality dimensions in an existing framework showed that the dimensions for secondary use of EHR data are applicable to person-generated wearable device data. There were 3 focus group sessions with domain experts in data quality and wearable device research. The experts concluded that intrinsic data quality features, such as conformance, completeness, and plausibility, and contextual and fitness-for-use data quality features, such as completeness (breadth and density) and temporal data granularity, are important data quality dimensions for assessing person-generated wearable device data for research purposes.

**Conclusions:**

In this study, intrinsic and contextual and fitness-for-use data quality dimensions for person-generated wearable device data were identified. The dimensions were adapted from data quality terminologies and frameworks for the secondary use of EHR data with a few modifications. Further research on how data quality can be assessed with respect to each dimension is needed.

## Introduction

### Use of Person-Generated Wearable Device Data for Research Purposes

The growing interest in quantified self along with the routine use of consumer wearables is generating substantial amounts of person-generated wearable device data [1,2]. These passively and objectively collected data hold great potential for use in biomedical research as they capture data that occur outside the clinic, without having to rely on patient recall [3]. An example of using wearable device data for biomedical research is a study by Lim et al [4] in which consumer-grade fitness tracker data (Fitbit Charge HR) was used along with survey and electronic health record (EHR) data. In addition, wearable device data can be reused in multiple studies to answer many different research questions. The investigators of the Lim et al [4] study made their data publicly available for other researchers, expanding the opportunity to generate and validate medical evidence. McDonald et al [5] used these data to investigate the relationship between sleep time and BMI in a Chinese population. This study was conducted to confirm the results of Xu et al [6], who examined the relationship between sleep duration and BMI. One of the limitations of the Xu et al [6] study was that their data primarily consisted of Europeans, and thus the study results needed further investigation to be generalizable. McDonald et al [5] added further evidence to the association between sleep and BMI by examining the same research question using a data set comprising Asian individuals. This type of evidence generation is expected to become more widespread with the All of Us Research Program, a precision medicine initiative by the National Institutes of Health, which is collecting, integrating, and providing wearable device data (eg, Fitbit) to the public for research purposes [7]. Considering that there is a lack of publicly available data sets generated from consumer wearable devices with a large number of participants and long-term observation, the All of Us data are expected to become a promising resource for many researchers interested in analyzing wearable device data.

### Significance of Data Quality Assessment

Although person-generated wearable device data are a promising new source of biomedical data, there are concerns regarding the quality of data. For example, missing data owing to users not wearing the device or incorrect data owing to device malfunction are a few data quality problems that could occur [8,9]. As these data anomalies could lead to various challenges when analyzing wearable device data, data quality assessment is a critical step that should be implemented before any analyses [8]. In this setting, data quality assessment is not only about whether the wearable device captures valid and reliable data but also whether a data set is fit-for-use for a specific research purpose, ensuring valid results [8,10]. However, the question about what data quality means, more specifically, how data quality is defined for the use of person-generated wearable device data for research purposes still remains.

### Data Quality Dimensions

Data quality dimensions are criteria or aspects of data quality that are considered essential for a specific user’s task and are constructs used when assessing data [11,12]. For example, the quality of data could be assessed in terms of its completeness (“Are data values present?”), conformance (“Do data values adhere to specified standards and formats?”), and plausibility (“Are data values believable?”) [13,14]. Various methods have been previously used to derive the data quality dimensions for biomedical data sets. First, Weiskopf et al [13] and Johnson et al [15] used systematic reviews to derive data quality dimensions. They both abstracted data quality attributes from studies on EHR data quality and then derived broad dimensions of data quality [13,15]. Second, stakeholder meetings are another method used by Kahn et al [14]. Stakeholders reviewed the literature on data quality, publications on best practices, operational manuals, and data quality rules from several EHR-based research networks. Data quality terms were then integrated into categories through an iterative process [14]. Finally, surveys have also been used as a method to identify data quality dimensions [16,17]. For example, Huang et al [17] investigated important data quality dimensions for genome annotation by asking genomic researchers to rank the importance of 17 data quality dimensions. The strength of these empirical methods is that it captures the perspective of data users and reveals data quality dimensions that may not have been considered by data quality researchers [16,18]. This is important as data quality is a concept that depends on the data users and their research tasks.

Currently, there is a lack of studies that derive dimensions for person-generated wearable device data using empirical methods. To our knowledge, the study by Codella et al [19] is the most relevant study on data quality dimensions for person-generated wearable device data. The study [19] first reviewed the literature to identify stakeholders’ concerns regarding person-generated health data (PGHD) and mapped the concerns to the corresponding data quality dimensions in the Wang and Strong [16] framework. However, the Wang and Strong [16] framework was derived by surveying business data consumers, which might not include important data quality dimensions for PGHD. Therefore, there is a great need to investigate the essential challenges and dimensions for assessing the quality of person-generated wearable device data for biomedical research because it is a growing, new data type.

### Objective

The aim of this study is to identify important data quality dimensions for using person-generated wearable device data for research purposes. The focus of this study is on intrinsic (data quality features inherent to the data) and contextual and fitness-for-use data quality dimensions (features that are task-dependent). Extrinsic and operational data quality features, such as data accessibility, security, or privacy, are not the focus of this study.

## Methods

### Study Design

Owing to the lack of literature or experts in the data quality field for person-generated wearable device data, a multi-method approach was used to complement and validate information found by each method. A combination of literature review and survey was used to improve reliability through constant data comparison [20]. In addition, focus group discussions were conducted to derive data quality dimensions from the collected data.

### Part 1: Literature Review

The goal of the literature review was to identify (1) factors affecting the quality of person-generated wearable device data and (2) associated intrinsic data quality challenges that could potentially occur when conducting research. Studies were examined from scholarly databases using a combination of search terms related to data quality and wearable devices. One reviewer (SC) screened the titles and abstracts of the studies based on a set of selection criteria. For example, studies containing any content on the data quality of wearable device data or sensor data when used for research purposes were included, but studies on clinicians wearing devices for patient care were excluded because the focus was on person-generated data being used for research purposes. The full text was screened using the same criteria by 2 reviewers (SC and KN). Sentences on data quality challenges and factors affecting those challenges were annotated, and semantically similar challenges and factors were grouped into the same category. The categorization process was performed by 3 researchers (SC, KN, and Ipek Ensari), including the 2 reviewers (SC and KN). Details of the literature review process are described in a previously published manuscript [9].

### Part 2: Survey

#### Survey Development

The survey was developed with a mixture of multiple-choice, open-ended, and Likert-type scale questions. The survey was iteratively refined based on feedback from 6 experts—3 in data quality, 2 in wearable devices, and 1 in survey development. The experts were recruited through the professional network of the research team, and the experts were those who actively conducted research in either data quality, wearable devices, or survey development. A web-based survey was created using Qualtrics (Qualtrics; version August 2019), which is a web-based survey software [21].

#### Data Collection and Analysis

The eligibility criteria for survey participation included the following: (1) an individual with experience in analyzing passively collected wearable device data for their research and (2) an individual with knowledge of data quality challenges when dealing with wearable device data. Potential survey participants were identified by searching the authors of research studies that used wearable device data and through referrals. The survey link was sent via email to the candidate respondents. In addition, a link to the survey was posted on the Observational Health Data Sciences and Informatics forum [22]. This forum was chosen because it focuses on observational health data, and individuals with diverse research backgrounds including PGHD and data quality frequently visit the forum. Participation was voluntary, and the survey was self-administered and anonymous.

Answers to multiple-choice questions were analyzed using descriptive statistics, and thematic analysis was conducted to identify themes from answers to open-ended questions. Responses to Likert-type scale questions were analyzed by comparing mean (importance of the dimensions) with SD (reliability) of the importance ratings of the dimensions. Dimensions with high mean (importance) and low SD (less variability in ratings among respondents) were determined as important.

### Part 3: Focus Group Discussion

Domain experts in data quality or wearable device data were recruited through a professional network of authors. The facilitator (SC) combined the results of the literature review and survey and proposed potential dimensions to domain experts. Domain experts discussed the information provided and determined whether to accept or reject the suggested data quality dimensions. The importance ratings on dimensions in the harmonized intrinsic data quality framework (HIDQF) were also used as a reference to determine its relevance to wearable device data [14]. The discussion continued until consensus was reached among the experts.

## Results

**Figure 1:**
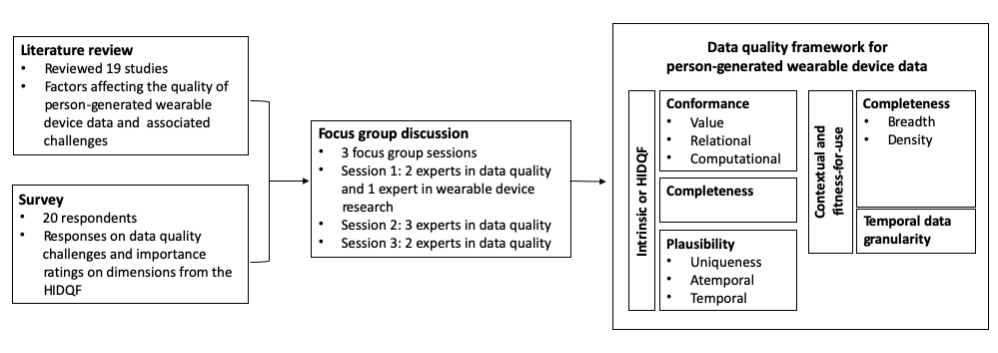
An overview of study processes and results. HIDQF: harmonized intrinsic data quality framework.

An overview of the results is depicted in Figure 1 followed by further details regarding the results.

### Part 1: Literature Review

In total, 1290 studies were retrieved and screened, resulting in 1.47% (19/1290) studies being selected for analysis. Data extracted from the studies were categorized into 3 groups of factors affecting data quality, that is, device- and technical-related, user-related, and data governance–related factors, and 3 data quality challenges, that is, incompleteness, incorrectness, and heterogeneity. Most studies have discussed device- and human-related factors that influence data quality. For example, device malfunction, network and connectivity, and users not wearing the device can lead to incomplete or missing data, whereas poor quality of sensor or algorithms and users’ incorrect use of the device may lead to incorrect data. In addition, lack of data standardization, such as different data formats, measurement units, and different algorithms, for the same parameter may cause difficulty in making a direct comparison between data from different devices. The full results of the literature review have been published [9].

### Part 2: Survey

#### Survey Design and Participant Recruitment

The survey was designed in 3 parts: (1) questions on the respondents’ research background, (2) questions on the research that the participants have conducted, and (3) questions on participants’ perception and knowledge of data quality. The survey included a Likert-type scale question that asked to rate the data quality dimensions from the HIDQF regarding their importance [14]. The HIDQF harmonizes 9 existing data quality terminologies and frameworks that are applicable to the secondary use of EHR data [14]. The harmonized framework involved a consensus among various stakeholders and experts in data quality; thus, it made sense to leverage the framework as a basis for the data quality dimensions of wearable devices. The full survey can be found in the link cited in the reference [23].

Emails were sent out to 100 researchers from August 2019 to September 2019. The exact number of survey recipients is unknown because the email recipients forwarded the email to other eligible individuals, and the survey was posted on a public online forum. In total, 20 responses were collected—most respondents were from the United States, but there were also a few respondents from the United Kingdom, France, and Singapore. Using 100 as a proxy for the number of eligible researchers, there was a 20% (20/100) response rate for the survey.

#### Background of Respondents

Table 1 shows the background of the survey respondents based on the responses collected from part 1 and part 2 of the survey.

Most respondents published 1-3 peer-reviewed articles (12/20, 60%), and 3 respondents (3/20, 15%) published >10 articles. The most common types of studies previously conducted by respondents were device validation or reliability studies (11/20, 55%), modeling to predict health state (10/20, 50%), and tracking behavioral changes (8/20, 40%). Other research types, such as pattern analysis on activity data and tracking body movement or stress, were also mentioned.

Nearly half of the respondents (9/20, 45%) used research-grade and consumer-grade devices with similar frequency, and 8 respondents (8/20, 40%) had only used consumer-grade devices. The respondents gave multiple answers regarding the brand and model of the devices they had used before. Among consumer-grade devices, the most frequently mentioned brand was Fitbit (19/20, 95%), followed by Garmin, Withings, Jawbone, and Apple Watch. Research-grade devices, especially accelerometers, such as ActiGraph, GENEactiv, and Actical, were mentioned 6 times. Other devices were mentioned, such as the Huawei Watch 2, Samsung Gear 2, and Misfit Shine 2.

**Table 1:** Background of respondents (N=20).

Characteristic		Value
**Number of peer-reviewed articles using wearable device data, n (%)**		
	None	1 (5)
	1 to 3	12 (60)
	3 to 5	2 (10)
	5 to 10	2 (10)
	10 or more	3 (15)
**Type of research conducted (multiple choice possible), n (%)**		
	Device validation or reliability studies	11 (55)
	Modeling to predict health state	10 (50)
	Modeling to inform treatment decisions	2 (10)
	Tracking behavioral changes	8 (40)
	Other	3 (15)
**Type of devices used for research, n (%)**		
	Consumer-grade wearable	8 (40)
	Research-grade wearable	3 (15)
	Used both with similar frequency	9 (45)
**Brand of devices used (multiple choice possible), n (%)**		
	Fitbit (Charge HR, Alta HR, Ultra, etc)	19 (95)
	Garmin (Vivofit, Vivosmart, Fenix, etc)	6 (30)
	Withings (Go, Pulse, or BP cuff)	4 (20)
	Jawbone (UP)	2 (10)
	Apple Watch	1 (5)
	Accelerometer (ActiGraph, GENEactiv, etc)	6 (30)
	Other (Huawei, Samsung gear, Misfit, etc)	14 (70)

#### Data Quality Challenges

In total 3 main themes and 1 minor issue were derived from the open-ended question on data quality challenges: (1) completeness, (2) accuracy, (3) plausibility, and (4) data access and semantics.

##### Completeness

One of the major themes was the completeness. Missing data were a concern for the respondents because of the uncertainty involved in dealing with missingness as it can have a negative effect on the analysis results. Many respondents wrote about missing data caused by various reasons, such as device error or users not wearing devices, which aligns with the results from the literature review. One respondent specifically talked about a different aspect of missing data, which is the lack of a certain variable that they needed for their research (*“*Lack of availability of heart rate variability*”*).

##### Accuracy

Another major theme was accuracy—*Do the data represent the true value?* Respondents talked about their doubts about whether the data correctly capture the true physiological measure they are supposed to represent. For example, steps might not be counted if one does not wear the device during exercise owing to discomfort. On the other hand, other activities, such as motorcycle rides, could falsely increase the step counts. In addition, a respondent mentioned the problem of GPS devices only recording known locations rather than the actual route, affecting distance traveled. There could also be inaccuracies in the sleep data. For instance, activities that are performed while lying on the bed (eg, using phones) could be counted as sleep mode, and sleep or wake time could be recognized inaccurately. These concerns match the challenges found in the literature review.

##### Plausibility

Plausibility was another major theme—*Do the data make sense?* One of the issues mentioned was that the data did not agree with their common knowledge. For example, there are problems in inconsistency between variables (“large spikes or drops in activity that are highly inconsistent with their surrounding measured values”). Respondents also stated that outliers in the data made them question the validity of that data point (“knowing whether unusual data are real”).

There were also time-related plausibility issues. For example, even though the data for 2 different variables are captured at the same time, the recorded timestamp on the server could be different between the 2 variables because of problems with data upload (“lag between device and data server—some variables are collected at slightly different time due to problems with wifi connection, data uploading”). In addition, people traveling between different time zones may produce implausible time patterns when the device does not recognize the change in time zone (“Subjects may travel between different time zones during study period. Some devices don't recognize a different time zone and the recorded data has weird time pattern that is hard to understand”). These challenges were not explicitly mentioned in the literature but are implied by incorrect data problems.

##### Data Access and Semantics

There were data quality challenges related to data access and semantics. For example, the difficulty in accessing raw data and minute-level data was mentioned by a few respondents. In addition, a few respondents mentioned that interpreting the data may be a challenge because of the lack of information on context and provenance (eg, no documentation of exposures). Lack of transparency owing to consumer devices being proprietary was also mentioned. These challenges were not mentioned in our literature review study on data quality challenges because the scope of research was only on intrinsic data quality challenges, but there were studies mentioning these challenges.

#### Ratings of Data Quality Dimensions

**Figure 2:**
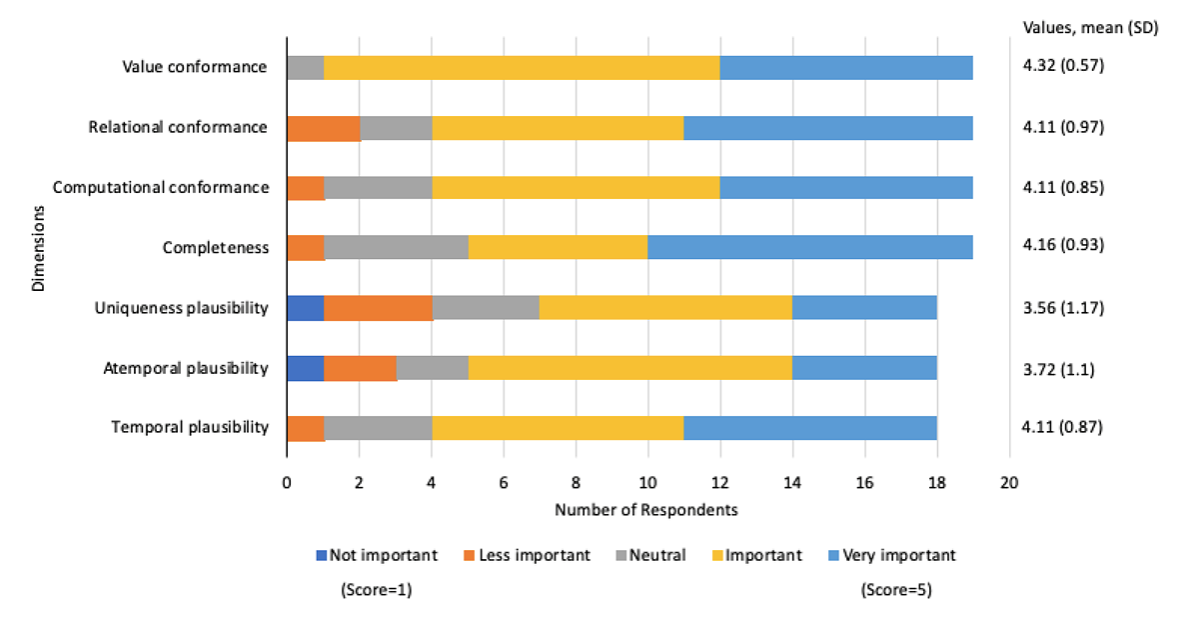
Importance ratings on dimensions from harmonized intrinsic data quality framework.

**Figure 3:**
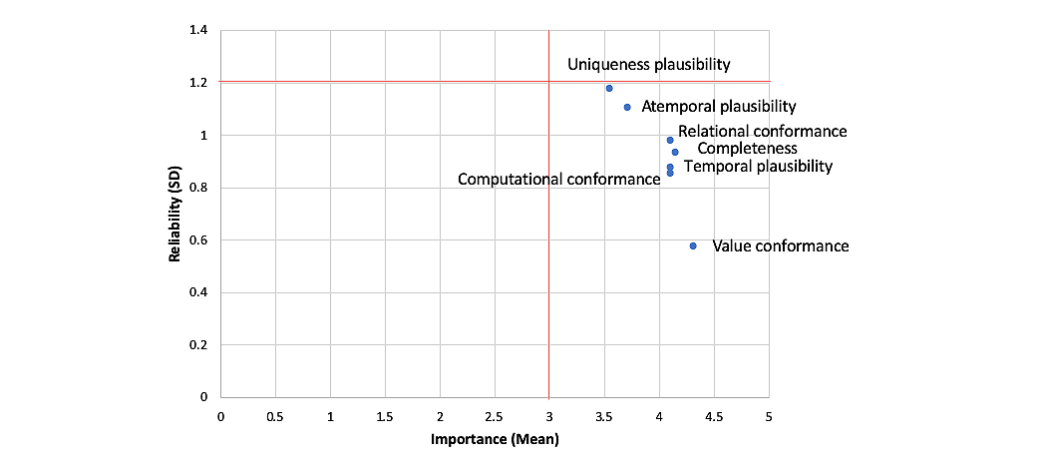
Importance versus reliability of ratings on data quality dimensions in the harmonized intrinsic data quality framework.

Respondents’ importance ratings on dimensions from the HIDQF are presented in Figures 2 and 3 [14].

Adopting the cutoffs used in a previously published study, dimensions with mean ratings>3 were determined to be important, and ratings with SD <1.2 were considered reliable [24]. Overall, respondents considered dimensions from the HIDQF as important data quality features for wearable device data. A follow-up question on the most important dimension identified completeness as the most important dimension (n=7), followed by relational conformance (n=4), computational conformance (n=4), value conformance (n=1), temporal plausibility (n=1), and atemporal plausibility (n=1).

A few respondents answered the free-response question on additional data quality dimensions that need to be added. Various problems were mentioned, including the importance of a consistent sampling rate when dealing with multiple device data and the need for contextual information about the data set. For instance, metadata on whether the data set is raw data or processed using proprietary algorithms and whether the users brought their own device or whether it was provided was considered important information to respondents. Furthermore, information on the wearing status of users was considered important.

### Part 3: Deriving Dimensions Through Focus Group Discussion

The potential data quality dimensions proposed by the facilitator (SC) are presented in Table 2 (the full version of this table can be found in Table S1 of Multimedia Appendix 1). Conformance was included as a potential data quality dimension based on the factors related to data heterogeneity found in the literature review and survey responses on the importance of data conformance. Completeness was one of the most frequently mentioned data quality challenge in both the literature review and survey. It was also selected as the most important data quality dimension by the survey respondents and thus was included in the list of potential data quality dimensions. Data quality challenges related to accuracy (data incorrectness) were frequently mentioned in both the literature and the survey. In addition, plausibility, which has a similar context with accuracy, was mentioned by survey respondents (eg, “large spikes or drops in activity that are highly inconsistent with their surrounding measured values”). Both challenges were presented to the experts for further discussion. The difficulty of accessing minute- or second-level data was mentioned as a challenge in both the literature and the survey (this is more of an extrinsic data quality challenge, which was why it was not reported in the previously published literature review study). As the objective of this study was to focus on intrinsic and contextual and fitness-for-use data quality dimensions, not extrinsic data quality dimensions, data accessibility was not included as a potential data quality dimension. Instead, the challenge of accessing minute- or second-level data was interpreted as the researchers’ need for more temporally granular data. Thus, temporal data granularity was added as a potential data quality dimension. Finally, data interpretability was proposed to domain experts based on survey responses on the need for contextual information and metadata.

**Table 2:** List of data quality dimensions suggested based on findings from literature review and survey.

Dimensions suggested to experts		Corresponding content from the literature review	Corresponding content from survey responses	Importance rating (only for HIDQF^a^)
**Conformance**				
	Value conformance	Different devices may use a different measurement unit.	“Data set not conforming to data dictionary will be hard to fix”	4.32
	Relational conformance	—^b^	“Without relational conformance you can't link one wearable device to another or to health outcomes”	4.11
	Computational conformance	Companies do not always reveal whether or when they update their device algorithms or whether or when the users install the provided software updates. Lack of standardization: (for multi-device studies) different devices may use different algorithms, a different definition for the same parameter, different sampling rate.	“I don’t know a way to proceed with the data analyses if the computational conformance isn’t met with satisfaction. it suggests that the data collected cannot be trusted.”	4.11
Completeness		Missing data due to various reasons: device malfunction, connectivity issues, nonadherence to the device, quality of skin contact of the device.	“Missing data is a large issue for our research, especially because we are trying to identify patterns or subsequences of activity. Missing data has to either be interpolated or treated as a zero value, and either of these methods can have a large negative effect on the results of our pattern mining techniques.”	4.16
Breadth completeness		—	“Lack of availability of HRV^c^”	
**Plausibility**				
	Uniqueness plausibility	—	—	3.56
	Atemporal plausibility	—	“Large spikes or drops in activity that are highly inconsistent with their surrounding measured values”	3.72
	Temporal plausibility	Companies do not always reveal whether or when they update their device algorithms, or whether or when the users install the provided software updates.	“Devices might cause problem with recording different time zone or time during traveling: Subjects may travel between different time zones during study period. Some devices don't recognize a different time zone and the recorded data has weird time pattern that is hard to understand”	4.11
Temporal data granularity		Fitbit only provides access to day-level data unless the minute-level or second-level data is requested and approved.	“Access to minute level data.”	—
Accuracy		Poor data accuracy caused by device malfunction, unknown limitations of proprietary algorithms, user error in device use.	“Other activities generating step counts (eg, motorcycle ride, vibration)” “Inaccurate sleep and wake time recognition”	—
Interpretability		—	“Trying to nail down exactly what a participant was doing when data was being collected offsite.”	—

^a^HIDQF: harmonized intrinsic data quality framework.

^b^No available data.

^c^HRV: heart rate variability.

**Figure 4:**
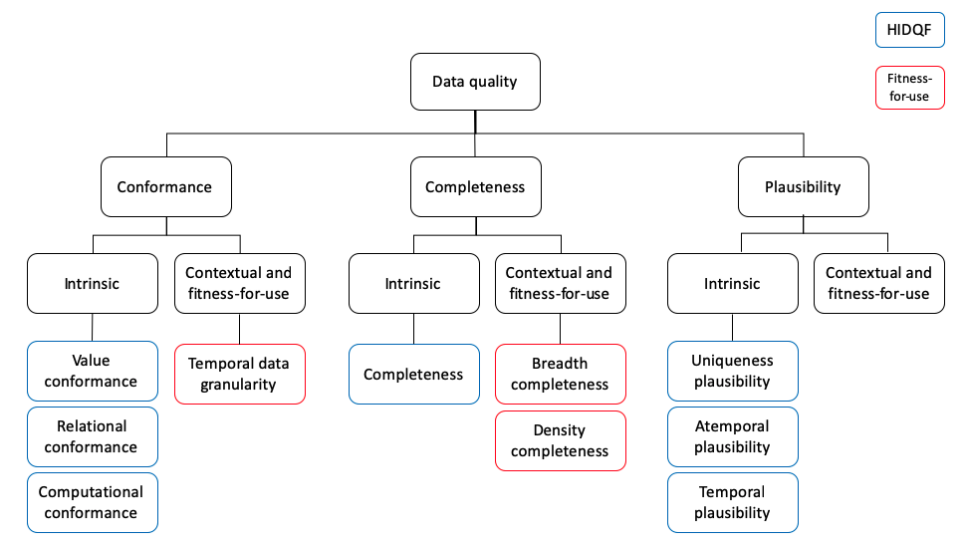
Data quality dimensions for assessing person-generated wearable device data for research purposes. HIDQF: harmonized intrinsic data quality framework.

In total, 3 separate discussion sessions were conducted in January, May, and September 2020. All sessions were conducted with 2-3 domain experts and 1 facilitator. In all, 2 data quality experts and 1 wearable device expert participated in the first discussion session. To continue the discussion on the relevance of dimensions to wearable device data, the second and third discussion sessions were conducted with 3 and 2 data quality experts, respectively. The domain experts agreed that all dimensions in the HIDQF were applicable to person-generated wearable device data. In addition, it was suggested to add contextual and fitness-for-use data quality dimensions that consider data quality in the context of a given research task [16]. Although the dimensions of the HIDQF are for research purposes as well, they focused on intrinsic data quality that assesses data quality in terms of the structure and presence of the data itself, independent of research tasks [14]. Considering that our focus was on using wearable device data for research purposes, aspects of data quality that can be determined once the research task is known were considered important. The final list of dimensions is shown in Figure 4.

There was substantial discussion on *completeness*. The completeness dimension in the HIDQF is defined as “Are data values present?” which measures completeness based on the presence of data without referring to research tasks [14,25]. However, determining missing data could be complicated for wearable device data when conducting research, especially for activity or step count data, because missing data could appear as null but more often as zero values [9]. Interpreting zero values is not easy because it could mean that a person was not wearing the device (true missingness) or was sedentary (a valid zero value). Zero values generated from being sedentary are not simply missing data, as they provide information on device users’ physical activity [26]. As it is impossible to know the cause of zero values, researchers typically make assumptions on thresholds for the inactivity period to determine nonwear time (eg, 60 minutes of inactivity [zero step count] is considered as a user not wearing the device) [26,27]. Thus, data completeness for activity-related data can be assessed based on the measures and thresholds that researchers set to define what is or is not missing data. This was why the fitness-for-use data completeness dimensions were considered important by domain experts. There were 2 fitness-for-use completeness dimensions determined as applicable to wearable device data, which were breadth and density completeness. Breadth completeness assesses whether a data set contains all types of data that are required for a specific task. For example, to investigate the association between activity and heart rate, a data set that does not provide heart rate data would not be suitable for use. Density completeness assesses whether a data set contains sufficient amount of data in terms of density, regularity, and duration. For instance, examining the association between step count and blood pressure might require the data set to have ≥10 days of step count data per month for 2 months [28]. The 2 subcategories of completeness, which are breadth and density completeness, were adopted from Weiskopf et al [25].

There was also a significant debate on whether accuracy (*Do the data reflect the true value?*) should be included as a dimension. On one hand, accuracy was considered a dimension that can be easily understood by stakeholders and the ultimate goal of data quality. On the other hand, accuracy was viewed as a vague term that could be interpreted in many different ways. For example, inaccuracy could be an umbrella term that incorporates invalid data, missing data, or data not conforming to data dictionaries. In addition, accuracy was considered inapplicable for assessing data quality from the secondary use of the data perspective. This is because it is impossible to know whether the data are correct or incorrect in the absence of a known truth. For instance, although the data indicated that an individual took 8 steps at 9 AM on April 5, 2020, there would be no way for a researcher to assess whether that is right or wrong when they retrospectively assess the accuracy of that value. The accuracy of the data values can only be assessed by comparing the device to a gold standard device. In reality, this is not feasible as people rarely wear more than one device in their daily lives, which restricts the ability to assess the accuracy of values in a longitudinal and continuously collected wearable device data. This was why the dimension *plausibility* (*Do the data make sense?*) was eventually included rather than *accuracy*.

Temporal data granularity was another fitness-for-use dimension considered important. As wearable device data are time-series data, the granularity of time points was deemed as an essential aspect. Temporal data granularity is about how frequently the data are documented (eg, every second, minute, or hour) and whether it fits the purpose of the research task. For example, a data set with timestamps every hour would not be suitable for research requiring data points every minute.

Other minor issues mentioned in the literature review and survey were not included as a dimension. For example, survey respondents mentioned the difficulty of interpreting data values, understanding what was really happening while data were being collected, or knowing how the data were collected. This was considered a metadata quality problem rather than a quality metric for the data. The definitions and examples of the final set of dimensions derived from focus group discussions are presented in Table 3.

**Table 3:** Data quality dimensions for person-generated wearable device data identified by domain experts.

Type and dimension				Definition^a^		Example
**Intrinsic**						
	**Conformance: Do data values adhere to specified standards and formats?**					
		Value conformance	Data values conform to internal formatting constraints, allowable values, or ranges.		Unit of distance is “miles.” “Sleep stages” only has values “deep,” “light,” “rem,” and “wake,” which conform to the data dictionary.	
		Relational conformance	Assuming there are multiple tables or files, recorded data elements agree with structural constraints imposed by the physical database structures that store data values.		Participant ID number links to other tables as required. The wearable device identifier is appropriately linked for all observations.	
		Computational conformance	Computations used to create derived values from existing variables yield the intended results either within a data set or between data sets.		Sleep duration conforms to the difference between start time and end time of sleep.	
	Completeness: Are data values present?			Missing data is determined based on the presence of data. Typically, absence of data is expected if the device is not worn, but this could sometimes be difficult to know retrospectively.		There is no NA (Not Available) in the step count data.
	**Plausibility: Are data values believable?**					
		Uniqueness plausibility	Objects do not appear multiple times in settings where they should not be duplicated or cannot be distinguished within a database or when compared with an external reference.		A single participant only has one participant ID number.	
		Atemporal plausibility	Observed data values, distributions, or densities agree with local or “common” knowledge or from comparisons with external sources that are deemed to be trusted or relative gold standards.		Step count and distance values are positive. Trends of step counts and distance agree with each other. Step counts do not show a sudden spike during sleep or during sedentary time. The range of heart rate values is biologically plausible. Heart rate is higher when active compared with when sedentary.	
		Temporal plausibility	Time-varying variables change values as expected based on known temporal properties or across one or more external comparators or gold standards.		Start time of sleep occurs before end time of sleep. Aggregate step count is higher during daytime than nighttime.	
**Contextual and fitness-for-use**						
	**Completeness: Are data values present fit for intended use?**					
		Breadth completeness	All data types required for intended use exist.		Heart rate data are essential for studies analyzing the relationship between physical activity and heart rate.	
		Density completeness	Data set contains a specified number of data values or occurs regularly over a certain period.		Heart rate should be measured at least once a day. Sleep data should be recorded every day consecutively for a 6-week period to be considered complete.	
	Temporal data granularity: does the device collect data granular enough for intended use?			Granularity of time stamps are sufficient for the task at hand.		Data values are recorded every second, which is appropriate for marathon research studies (the exact start and end time of the marathon for each runner is important for marathon-related studies).

^a^Definitions were adopted and adapted from the studies by Weiskopf et al [25] and Kahn et al [14].

## Discussion

### Principal Findings

In this study, data quality dimensions for person-generated wearable device data were identified using multiple methods. A literature review and survey was conducted to understand the data quality challenges of researchers and their perceptions on data quality dimensions. On the basis of this information, domain experts determined the appropriate dimensions. Experts agreed that the data quality dimensions from the HIDQF are applicable to person-generated wearable device data, and fitness-for-use dimensions were also considered important, especially for research purposes. The final data quality dimensions deemed important were intrinsic data quality dimensions, such as conformance, completeness, and plausibility, and fitness-for-use data quality dimensions, such as breadth and density completeness and temporal data granularity.

### Data Quality Assessment Guidelines for Researchers

#### Completeness

In this study, breadth and density completeness, which are contextual and fitness-for-use data quality dimensions, were considered important for conducting research. Assessing breadth completeness is important, especially for data sets collected in a bring-your-own-device research setting [9]. This is because different brands and models that users bring may collect different data types, which means that not all individuals in the data set would have all the data types that are needed to answer a research question.

Density completeness is also an essential fitness-for-use dimension for wearable device data because the amount of data sufficient and valid for a specific research task is determined by researchers. Researchers first need to determine how wear versus nonwear of the device is defined. Typically, consumer wearables do not provide information on the wear status; thus, researchers need to make decisions based on existing data. The recorded zero step counts could be due to nonwear (missing data) or it could mean inactivity, and thus researchers need to determine thresholds to define nonwear. An alternative method to determine the wearing status could be based on the existence of heart rate data or the values of heart rate data. For example, Lim et al [4] used the confidence values of heart rate data points as surrogate measures for which *−1* indicates invalid data because the device is not worn or incorrectly worn. This approach opens up the discussion on missing data, whether it should be simply based on the absence of data values or whether the default values for missing data and their semantic meaning should be considered. This was the reason why the fitness-for-use completeness dimensions were considered important.

On the basis of decisions made on wear versus nonwear, researchers can determine the appropriate level of data density for their research. Researchers can first determine the thresholds for how much health behavior data are sufficient for a day. For example, Tang et al [9,29] systematically addressed the incompleteness of physical activity data by presenting heuristic criteria for the definition of a valid day: a day is valid (1) if the step count is above a certain threshold, (2) if the number of hours with data is above a certain threshold, (3) if there are data within 3 periods. Researchers can also define completeness based on the number of valid days needed within a certain data collection period, or how regularly the data should be present for the individual data to be included in the analysis [9]. As recently released devices have the ability to examine various data types and collect data seamlessly for years, further investigation is needed to determine how completeness is characterized in research studies.

#### Conformance

Value, relational, and computational conformance are all considered important dimensions for wearable device data, but there are challenges in data management and quality assessment. Value and relational conformance can only be assessed in terms of the data dictionary and relational model specific to the brand, model, and version of the device but only if this information is publicly available. In addition, computational conformance can be assessed for values that can be calculated using generic equations, such as sleep duration, which is the difference between the start and end of sleep time. However, it can be difficult to assess computational conformance for variables calculated using proprietary algorithms, as these are not disclosed to data users. Another challenge related to data conformance is the lack of a common data standard for wearable device data. A common data standard would be crucial for a data set collected from disparate devices (eg, Apple Watch and Fitbit Charge HR), such as data collected under a bring-your-own-device protocol. There is a movement in the mobile health community, called Open mHealth, to create a common data schema that explicitly states the format and data definitions for patient-generated data [30]. Adopting these standards for wearable device data might solve the discrepancy between the definition of data values among multi-device data. For example, currently there is no industry standard for defining activity intensity (eg, light, moderate, and vigorous). These challenges indicate that facilitating the use of consumer wearables for research purposes would not be feasible without the support of device companies and the research community.

#### Plausibility

Plausibility aligns with the needs of researchers for accurate data values. For instance, data may be deemed implausible when step counts are higher than normal, but the corresponding heart rate values are lower than usual. Typically, researchers arbitrarily come up with their own rules to assess the plausibility of data before proceeding with the analysis. However, domain knowledge and a considerable amount of experts’ time are required to formulate a set of potential data quality rules. Thus, creating a knowledge base of data quality rules for person-generated wearable device data would not only save time for future researchers but also prevent the use of ad hoc data quality rules [9]. Another challenge for plausibility is that there are few known external benchmarks that can be used to validate or triangulate the data (data quality validation per the HIDQF). For example, the summary statistics of steps, active minutes, and BMI have been compared with the corresponding values in the Centers for Disease Control and Prevention survey (eg, Behavioral Risk Factor Surveillance System) [31]. Further discussion among the researcher community would be needed to find potential methods or data sources to check the plausibility of data.

Although plausibility was chosen over accuracy as a data quality dimension, it is true that many people are concerned about whether data values are trustworthy. Even though accuracy cannot be assessed in the secondary use of data scenarios, it could be indirectly verified through the results of device validation studies [32-34]. Thus, it is important to provide metadata information on the device brand, model, and version that generated the data set as each element can change device validity [35]. However, knowing the validity and reliability of a device is insufficient to understand the accuracy of data because there are other factors that affect data quality such as incorrect device use by the user. In addition, device validation studies are generally conducted in a controlled setting for a short period.

### Limitations

This study has a few limitations. First, the study focuses only on intrinsic and fitness-for-use data quality dimensions and thus does not include extrinsic data quality features, that is, features that affect the data but are not about the data values themselves (eg, security, privacy, or data accessibility). There might be contextual information or metadata that are considered important when determining the fitness-for-use of a data set. For example, some researchers might want to know the process or operational aspects of data collection (eg, Were the data collected under the bring-your-own-device policy or were devices provided?) [36]. These factors were not captured as a data quality dimension, but it is an aspect that might need to be considered when assessing the fitness-for-use of a data set. Second, the study was conducted with a small number of survey respondents and domain experts. Therefore, survey responses and experts’ opinions may not be representative and comprehensive. As survey responses match the results of the literature review, it is likely that the survey was able to capture most of the data quality challenges despite the small number of respondents. Furthermore, the intrinsic data quality dimensions identified in this study leveraged the dimensions of the HIDQF. The HIDQF was determined through iterative meetings with stakeholders and data quality experts; thus, it is highly likely that most intrinsic data quality dimensions were included in our final list of dimensions. In future studies, contextual and fitness-for-use data quality dimensions could be further investigated with a larger group of stakeholders of person-generated wearable device data.

### Conclusions

Person-generated wearable device data are an emerging data type for biomedical research because of the growing use of wearable devices in people’s daily lives. However, there is a lack of agreement on how data quality should be assessed for person-generated wearable device data. As the first step to solve this challenge, data quality dimensions were identified specifically for person-generated wearable device data. We found that data quality dimensions for secondary use of EHR data are applicable to person-generated wearable device data. The identified dimensions will be able to provide guidance to researchers on how data quality is defined and what aspects of data quality should be assessed for person-generated wearable device data. Further research on how data quality can be assessed with regard to dimensions is needed.

## Appendix

Multimedia Appendix 1 Dimensions suggested by facilitator and final decision by domain experts.

## Abbreviations

EHR: electronic health record
HIDQF: harmonized intrinsic data quality framework
PGHD: person-generated health data
