# Supplementary material for: Identifying Data Quality Dimensions for Person-Generated Wearable Device Data: Multi-Method Study
Source: JMIR Mhealth Uhealth. 2021 Dec 23;9(12):e31618. doi: 10.2196/31618 (PMC8738984; doi:10.2196/31618)
Supplement: Multimedia Appendix 1 [file mhealth_v9i12e31618_app1.docx]

**Table S1. Dimensions suggested based on results from literature review and survey, and decisions made by experts**

| **Dimensions Suggested to Experts** | | **Literature Review** | **Survey responses** | **Dimensions Importance Rating** | **Focus Group Result** |
| --- | --- | --- | --- | --- | --- |
|  | |  |  |  |  |
| **Conformance** | |  |  |  |  |
|  | **Value conformance** | different devices may use different measurement unit | "Dataset not conforming to data dictionary will be hard to fix" | 4.32 | Should be included |
|  | **Relational conformance** |  | "Without relational conformance you can't link one wearable device to another or to health outcomes" | 4.11 | Should be included |
|  | **Computational conformance** | (1) companies do not always reveal whether or when they update their device algorithms. Or whether or when the users install the provided software updates (2) Lack of standardization: (in the case of multi device studies), different devices may use different algorithms, different definition for the same parameter, different sampling rate | "I have been involved in studies where, a couple of years into the analysis, it turned out that vital outcome definitions in the data dictionary were wrong."  "i dont know a way to proceed with the data analyses if the computational conformance isnt met with satisfaction. it suggests that the data collected cannot be trusted." | 4.11 | Should be included |
| **Completeness** | | Missing data due to various reasons: Device malfunction, Connectivity issues, Non-adherence to the device, Quality of skin contact of the device | “Missing data due to device breakage, user error”  “Missing data : Data can be missing due to i)subjects forgetting to put the device back on every morning, ii) poor wifi connection, iii) researchers' decision to ignore certain portion of data since it has weird/abnormal pattern. It is hard to decide what to do with the missing data”  “Missing data is a large issue for our research, especially because we are trying to identify patterns/sub-sequences of activity. Missing data has to either be interpolated or treated as a zero-value, and either of these methods can have a large negative effect on the results of our pattern mining techniques.”  ". If data is frequently missing, it impacts the performance of the mining and clustering algorithms we use, and also affects the quality of the patterns discovered (Bias towards constant patterns or less-complex patterns that are not as useful for research purposes)." | 4.16 | Should be included |
|  | **Breadth completeness** |  | “Lack of availability of HRV” |  | Should be included |
| **Plausibility** | |  |  |  |  |
|  | **Uniqueness plausibility** |  |  | 3.56 | Should be included |
|  | **Atemporal plausibility** |  | “large spikes or drops in activity that are highly inconsistent with their surrounding measured values”  “Knowing whether unusual data are "real” is difficult”  "If observed data value does not agree with the common knowledge or gold standard, then it is hard to believe the measure I am using and the analysis results based on it are reliable/trustworthy." | 3.72 | Should be included |
|  | **Temporal plausibility** | (1) companies do not always reveal whether or when they update their device algorithms. Or whether or when the users install the provided software updates | ”Devices might cause problem with recording different timezone/time during traveling : Subjects may travel between different time zones during study period. Some devices don't recognize a different time zone and the recorded data has weird time pattern that is hard to understand..”; “Lag between device and data server - some variables are collected at slightly different time due to problems with wifi connection, data uploading, etc and it is hard to decide how to handle this issue”; "if using wearable data as relative to symptoms or contextual information, time-stamping must be accurate" | 4.11 | Should be included |
| **Temporal Data Granularity*** | | Fitbit only provides access to day-level data unless the minute-level or second-level data is requested and approved. (need for a marathon study) | “Access to minute level data.” |  | Should be included |
| **Device Accuracy** | | Poor data accuracy caused by: Device malfunction, Unknown limitations of proprietary algorithms, User error in device usage. | “Device on specific body location affects accuracy (e.g. wearing the device on your wrist is a problem when using strollers/carts/wagons)”  “Other activities generating step counts (e.g. motorcycle ride, vibration)”  “Mapping known places rather than showing the actual route”  “Activities done while lying on the bed is counted as sleep mode”  “Inaccurate sleep/wake time recognition”  “Slow response to heart rate change” |  | Should not include (Reason explained in main manuscript) |
| **Interpretability** | |  | “participant documentation of exposures” “trying to nail down exactly what a participant was doing when data was being collected offsite.” |  | Should not include  ("That’s more contextual knowledge. I would say that’s more ***metadata completeness***. I don’t know if that warrants a full data quality metric for the data itself.”) |
